# Simulation of Land Use Pattern Based on Land Ecological Security: A Case Study of Guangzhou, China

**DOI:** 10.3390/ijerph19159281

**Published:** 2022-07-29

**Authors:** Lesong Zhao, Guangsheng Liu, Chunlong Xian, Jiaqi Nie, Yao Xiao, Zhigang Zhou, Xiting Li, Hongmei Wang

**Affiliations:** 1 School of Public Administration, South China Agricultural University, Guangzhou 510642, China; zhaolesong@stu.scau.edu.cn (L.Z.); gsliu@scau.edu.cn (G.L.); chlxian@scau.edu.cn (C.X.); njq0506@stu.scau.edu.cn (J.N.); xiaoyaogo@stu.scau.edu.cn (Y.X.); zhouzhigang@stu.scau.edu.cn (Z.Z.); xitingli@stu.scau.edu.cn (X.L.); 2 Guangdong Province Key Laboratory of Land Use and Consolidation, Guangzhou 510642, China; 3 Key Laboratory of Natural Resources Monitoring in Tropical and Subtropical Area of South China, Guangzhou 510700, China

**Keywords:** land use pattern, land ecological security, scenario simulation, CA–Markov model, Guangzhou

## Abstract

The process of rapid urbanization has intensified the conversion of different land use types, resulting in a substantial loss of ecological land and ecological security being threatened. In the context of China’s vigorous advocacy of an ecological civilization, it is important to explore future land use patterns under ecological security constraints to promote sustainable development. The insufficient consideration of land ecological security in existing land use pattern simulation studies makes it difficult to effectively promote improvement in the ecological security level. Therefore, we developed a land use simulation framework that integrates land ecological security. Taking the sustainable development of land ecosystems as the core, the land ecological security index (LESI) and ecological zoning (EZ) were determined by the pressure–state–response (PSR) model and the catastrophe progression method (CPM). Natural development (ND) and ecological protection (EP) scenarios were then constructed taking the LESI and EZ into consideration. The CA–Markov model was used to simulate the land use pattern of Guangzhou for 2030 under the two scenarios. The results showed that (1) the study area was divided into four categories: ecological core zone, ecological buffer zone, ecological optimization zone, and urban development zone, with area shares of 37.53%, 31.14%, 16.96%, and 14.37%, respectively. (2) In both scenarios, the construction land around the towns showed outward expansion; compared with the ND scenario, the construction land in the EP scenario decreased by 369.10 km^2^, and the woodland, grassland, and farmland areas increased by 337.04, 20.80, and 10.51 km^2^, respectively, which significantly improved the ecological security level. (3) In the EP scenario, the construction land in the ecological core zone, ecological buffer zone, and ecological optimization zone decreased by 85.49, 114.78, and 178.81 km^2^, respectively, and no new construction land was added in the ecological core zone, making the land use pattern of the EP scenario more reasonable. The results of the study have confirmed that the land use pattern simulation framework integrating land ecological security can effectively predict land use patterns in different future scenarios. This study can provide suggestions and guidance for managers to use in formulating ecological protection policies and preparing territorial spatial planning.

## 1. Introduction

Land use/cover change is the main cause, and an important part, of global environmental change; is the result of the interaction between human activities and the natural environment, directly affecting the value and function of ecosystem services; and is key to ensuring the sustainable development of land ecosystems and maintaining ecological security [1,2]. Over the past decades, the dramatic increase in population and the rapid progress of industrialization and urbanization have led to a series of ecological problems, such as soil erosion, land desertification, habitat fragmentation, and loss of biodiversity [3,4,5], restricting the sustainable use of resources and sustainable economic and social development [6]. In this context, optimizing land use patterns and ensuring regional ecological security have become the focus of attention in the study of ecological environment change and sustainable development and play an important role in the study of global ecosystem change and socio-economic development [7,8].

Land use pattern is a direct reflection of the interaction between a socio-economic system and ecosystem at the temporal and spatial scales, involving complex processes and mechanisms, and is a prerequisite and basis for analyzing economic development and ecological environmental protection [9,10]. In-depth exploration of the competition process and evolutionary mechanisms among land types and systematic, multi-factor, and typical scenario land use pattern simulation studies can help optimize land use patterns and improve regional ecological security [11,12]. Many scholars have carried out extensive exploratory research on land use pattern simulations using different simulation models and have achieved relatively rich research results. At present, the common land use simulation models mainly include three types. The first is quantitative prediction models, including the Markov [13] and system dynamics (SD) models [14], which are mainly used to describe the structural and quantitative characteristics of the land use change process and cannot describe changes at spatial scales. The second is spatial models, such as the cellular automata (CA) model [15]; the agent-based model (ABM) [16]; and the slope, land use, exclusion, urban extent, transportation, and hill shade (SLEUTH) model [17]. The CA model focuses on the influence of environmental suitability on land use patterns, but the transformation rules are difficult to determine and are usually combined with other models to improve simulation accuracy; the ABM determines rules according to subject characteristics, which are not universal; and the SLEUTH model is a simulation model developed based on the CA model, with the advantages of simple operation and an open-source environment, but less consideration of the impact of socio-economic factors [18]. Third is the integrated model; however, it is difficult to integrate the complex change characteristics of land use simulation with a single model, so most scholars use comprehensive or improved models for land use pattern simulation [19], such as the Markov–FLUS model [20], the CLUE–S model [21], the CA–Markov model [22], etc. Based on the suitability atlas and transfer probability matrix, the CA–Markov model couples the ability of the CA model to simulate complex spatial and temporal variability with the advantages of the Markov model for long-term prediction and is widely used in land use pattern simulation [23]. However, it cannot explain the driving factors of land use pattern evolution or determine their importance. Multi-criteria evaluation (MCE) is an effective tool for use in decision making that analyzes the factors affecting the target [24]. Therefore, this study used the binary logistic regression (BLR) method to determine the driving factors and weights of land use pattern evolution in different periods and constructed the CA–Markov model integrating BLR and MCE for future land use pattern simulation.

As the basic carrier of material, land use pattern changes have an important impact on ecological security. With the intensification of the contradiction between humans and the land, ecological security in land use has become increasingly prominent, and land ecological security has attracted considerable attention, becoming an important field of research [25]. Most studies have focused on analyzing current or past ecological security patterns and proposing optimization strategies, while few studies have combined ecological security patterns with land use pattern simulations, as they have not been adequately considered. Research on combining ecological security patterns with land use patterns has been carried out from three main aspects. The first is to directly use rivers and scenic areas as spatial restrictions and to prohibit use conversion [26], a simple approach that can easily result in the omission of important ecological regions. The second approach is to identify important ecological areas based on models and use them as restrictions; for example, Yang et al. constructed ecological security patterns based on the morphological spatial pattern analysis (MSPA) method and a minimum cumulative resistance (MCR) model and used them as restrictions [27]. This approach takes more into account the natural environmental factors and less into account the socio-economic factors. Third, the future ecological security is assessed by the predicted land use pattern [7]. This method only judges the ecological level based on the land use type and lacks a comprehensive consideration of the evolution of ecological conditions. Most studies only regard important ecological areas as the limiting factors, without considering the suitability of various land conversions in different levels of ecological areas, while the selection of driving factors for the evolution of the land use pattern is not comprehensive enough, and the weight setting is subjective. 

Land-based rapid urbanization is an important mechanism enabling China’s rapid economic development. From 2000 to 2020, China’s urbanization rate increased from 36.22% to 63.89%, with urban construction land increasing by 1.64 times (data from the *China Urban Construction Statistical Yearbook*). The contradiction between land use and ecological protection has become more prominent with the increasing demand for urban scale and construction land. China is in a critical period of transition from rapid economic growth to high-quality development. The Chinese government is very focused on ecological protection and views ecological civilization construction as an important part of the national strategic layout. With the establishment of the urban agglomeration of the Guangdong–Hong Kong–Macao Greater Bay Area, the Pearl River Delta is under great pressure from population influx as well as limited resources and environment carrying capacity. Guangzhou is an important urban development area, population agglomeration area, and ecological security protection zone in the Pearl River Delta region, which occupies a very important position in economic development and ecological security pattern construction. Rapid economic development has resulted in great changes in land use patterns, intensified constraints on land ecosystem carrying capacities, and posed great threats to ecological security. The *Transforming our World: The 2030 Agenda for Sustainable Development*, adopted by the United Nations in 2015, sets the 2030 Sustainable Development Goals (SDGs), one of which is to protect, restore, and promote the sustainable use of terrestrial ecosystems. Land use patterns are closely related to the sustainable development of terrestrial ecosystems; as one of the first cities to respond to this goal, Guangzhou urgently needs to optimize land use patterns to achieve sustainable goals. Therefore, exploring the land use pattern under the constraints of ecological security in Guangzhou has a certain guiding significance for the protection of ecological security in China’s rapid development areas. In view of the above, a land use pattern simulation framework integrating land ecological security was proposed based on the land ecological security (LES) evaluation and the CA–Markov model. The LES evaluation classifies ecological zoning through a land ecological security index and determines the ecological security level in the study area. The integrated land use pattern simulation framework is able to achieve scenario simulation under ecological security constraints, which is of great significance for promoting regional sustainable development and optimizing land spatial patterns. The objectives of this study are to (1) assess the ecological security level of land and delineate ecological zoning; (2) predict the land use pattern for 2030 in natural development scenarios and ecological protection scenarios; and (3) analyze the changes in land use patterns under different scenarios by comparing the simulation results and propose optimization strategies.

## 2. Study Area and Data Sources

### 2.1. Study Area

**Figure 1:**
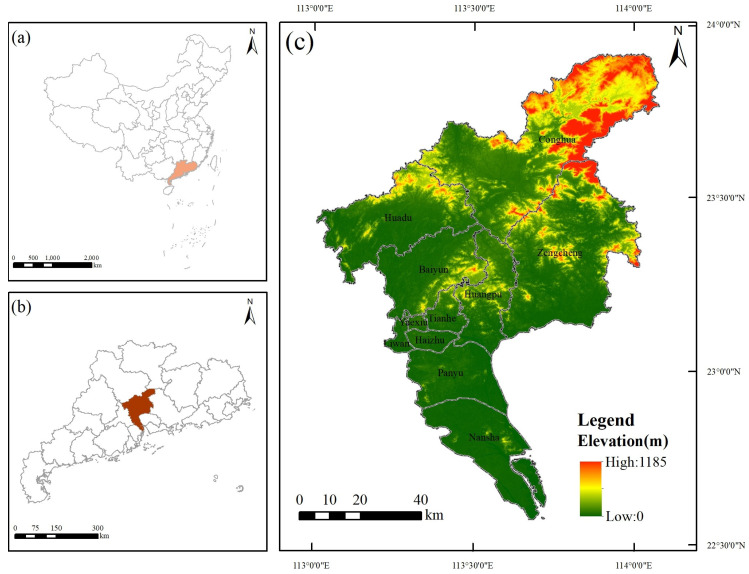
Location of the study area: (**a**) Guangdong’s location in China; (**b**) Guangzhou’s location in Guangdong; (**c**) Administrative and topographic map of Guangzhou.

Guangzhou is located in the south-central part of Guangdong Province (112°57′–114°03′ E, 22°26′–23°56′ N), at the north-central end of the Pearl River Delta, bordering the South China Sea, and has a total area of 7434.40 km^2^ (Figure 1). The topography of Guangzhou is high in the northeast and low in the southwest, and the terrain is complex and varied, with low and medium mountainous areas in the northeast, hilly basins in the center, and alluvial plains in the south of the Pearl River Delta. Guangzhou has a maritime subtropical monsoon climate, characterized by warm and rainy conditions, abundant light and heat, and an average annual temperature of 20 °C–22 °C, with rich hydrological and biological resources and superior natural endowments. In the past two decades, Guangzhou has grown rapidly, with the urbanization rate increasing from 62.14% to 86.19%. By the end of 2020, the total resident population of Guangzhou was 18.740 million, an increase of 11.807 million compared with 2000, which exerted great pressure on resources and the environment. In recent years, in response to the strategy of ecological civilization construction proposed by the central government, Guangzhou municipal government has issued a series of plans and documents, such as the overall urban environmental planning and the outline of the ecological civilization construction planning, which indicate the need to adhere to ecological priority and promote high-quality development. As the core engine of development in the Guangdong–Hong Kong–Macao Greater Bay Area, working out how to configure future land use to achieve sustainable development while reconciling the conflict between economic development and the environment is an important issue that Guangzhou needs to address.

### 2.2. Data Cources

The data were divided into geospatial data and socio-economic data. The geospatial data included land use raster data with a resolution of 30 m for the years 2000, 2010, and 2020, which were obtained from the Resource and Environment Science and Data Center of the Chinese Academy of Sciences (https://www.resdc.cn/, accessed on 26 October 2021). The land use types were classified into six categories: farmland, woodland, grassland, water area, construction land, and unused land through reclassification; rivers, towns, and rural settlements were extracted from these data. The digital elevation model (DEM) data were sourced from the Geospatial Data Cloud website (https://www.gscloud.cn/, accessed on 18 November 2021) with a spatial resolution of 30 m and were used to calculate elevation and slope. The population spatial raster data were drawn from Worldpop datasets (https://www.worldpop.org/, accessed on 18 November 2021) and included two phases from 2010 and 2020, with a resolution of 1 km. Information regarding highways and permanent basic farmland was extracted from land use vector data, which were sourced from the Guangzhou Natural Resources Department. In order to maintain a consistent raster scale in the simulation, the raster data were resampled to 30 m. The socio-economic data were collected from the *Guangzhou Statistical Yearbook* (2021), the *Guangdong Rural Statistical Yearbook* (2021), statistical yearbooks of each district, and statistical bulletins on national economic and social development, as well as from information published on the websites of government departments.

## 3. Methodology

**Figure 2:**
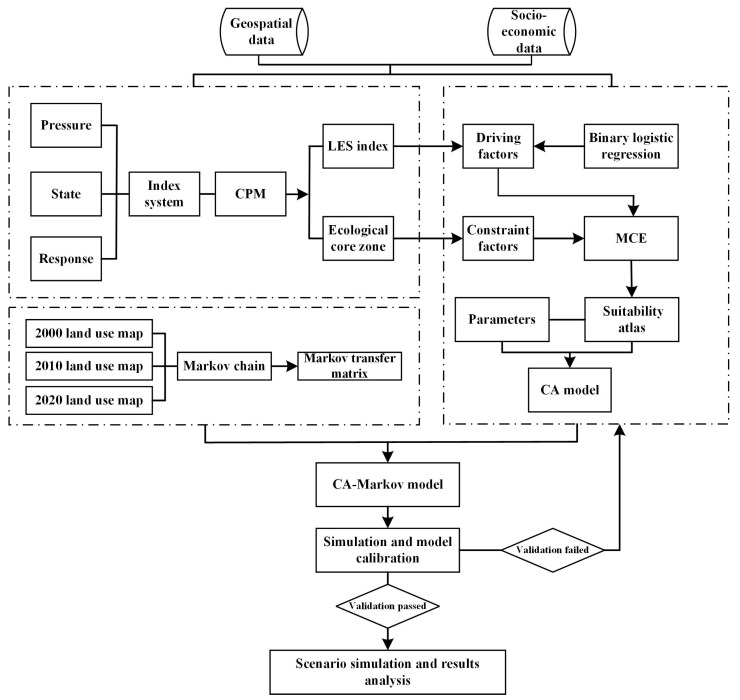
The research framework of land use simulation integrating land ecological security (LES).

The research framework is shown in Figure 2 and consists of three main parts: (1) starting from the aspect of land ecological security, an index system was constructed based on the pressure–state–response (PSR) model with a 1 km grid as the unit, and the catastrophe progression method (CPM) was applied to calculate the land ecological security index and divide the ecological zoning; (2) a simulation model of future multi-scenario land use patterns coupled with land ecological security was built using the CA–Markov model based on the multi-criteria evaluation (MCE) and the binary logistic regression (BLR) model; and (3) land use patterns in natural development and ecological protection scenarios were simulated, and the simulation results were analyzed.

### 3.1. Land Ecological Security Evaluation

#### 3.1.1. Catastrophe Progress Method

The catastrophe progress method (CPM) is a comprehensive evaluation method based on the catastrophe theory, which is based on the principle that the values of control variables falling within the bifurcation set can cause mutation in the state of the evaluation object and is suitable for systems with complex internal hierarchy [28]. The method takes into account the relative importance of indicators and reduces the influence of weight assignment on the evaluation results, thus reducing subjectivity without losing rationality [29]. The steps of the CPM applied to land ecological security evaluation are as follows. (1) Decompose the total objective in multiple layers according to the recursive structure and rank indicators at the same layer and with the same characteristics according to their importance. (2) Determine the catastrophe model of each layer. Catastrophe models consist of state variables and control variables. The number of state variables in common catastrophe models is only one, and the number of control variables is generally not more than four. According to the number of state variable decompositions, catastrophe models are mainly divided into folding model, cusp model, swallowtail model, and butterfly model (Table 1). (3) Calculate subordinate function values. Using the normalization formulas of the catastrophe model, according to the complementary principle, the values of the state variables of each layer are calculated, and the total membership function values are obtained from a down to up recursive calculation. Among them, the complementary principle means that there is a significant correlation between the control variables at all layers; while the non-complementary principle means that there is no significant correlation between the control variables at all layers.

#### 3.1.2. Establishment of Evaluation Index System Based on PSR Model

**Figure 3:**
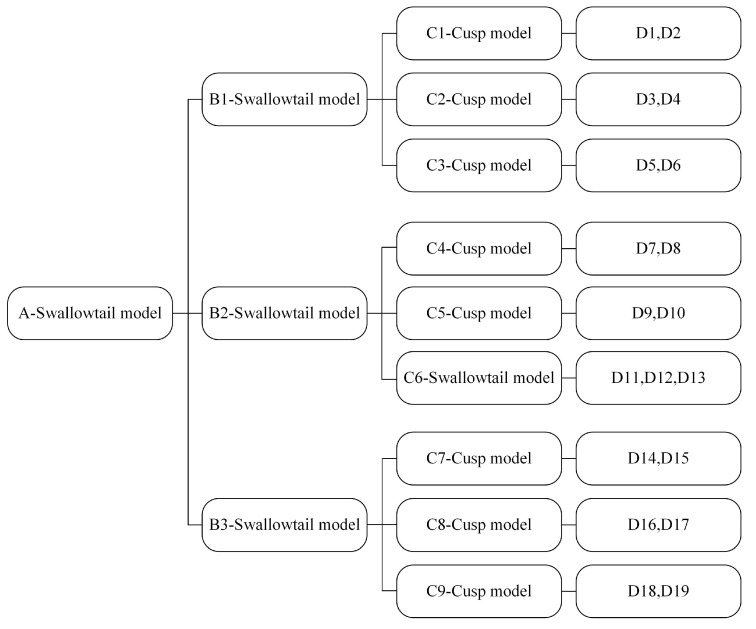
Catastrophe progress model integrating with LES.

The PSR model is a theoretical framework proposed by the Organization for Economic Cooperation and Development (OECD) and the United Nations Environment Programme (UNEP) to reflect the interrelationship between human activities and the ecological environment, and it is widely used in ecosystem health evaluation research [30]. Of these, pressure indicators measure the pressure of human activities on the environment; status indicators reflect the current state of the ecosystem; and response indicators stress a series of measures taken by human beings to improve the level of ecological security [31,32]. Based on the PSR model and considering natural and socio-economic factors, we selected 19 indicators by referring to the relevant literature [6,8,33,34] (Table 2). LES evaluation based on the catastrophe progress method requires the ranking of indicators at each layer, and in order to reduce the subjectivity of the assignment weight, our study used the entropy weight method to determine the importance of indicators and then determined the catastrophe model integrating land ecological security (Figure 3).

#### 3.1.3. Standardization of the Index

To eliminate the effects of different kinds of indicators and make the data comparable, we transformed the socio-economic data and geospatial data into grid units by intersection and spatial connection and standardized the indicators using the standardization method, which was calculated as follows:

(1) $$\mathrm{Positive}\;\mathrm{index}:\;H_{ij}=\frac{x_{ij}-min\left(x_{ij}\right)}{max\left(x_{ij}\right)-min\left(x_{ij}\right)}$$

(2) $$\mathrm{Negative}\;\mathrm{index}:\;H_{ij}=\frac{max\left(x_{ij}\right)-x_{ij}}{max\left(x_{ij}\right)-min\left(x_{ij}\right)}$$

where *H_ij_* represents the normalized value of the *j*th index in the *i*th grid; *x_ij_* represents the actual value of the *j*th index in the *i*th grid; and *min*(*x_ij_*) and *max*(*x_ij_*) represent the minimum and maximum values of the *j*th index in the whole region, respectively.

#### 3.1.4. Determination of Classification Criteria

Due to the high comprehensive index calculated by the catastrophe progression method, it is difficult to determine a reasonable grade range using the conventional equal grading method. In order to make the grade classification more differentiated, our study referred to Song et al. [35] and used the following method to improve the evaluation results: first, the affiliation value *n_i_* (*i* = 0, 1, 2, …, 7, 8, 9) was calculated for each indicator at the index layer as {0, 0.1, 0.2, …, 0.9, 1.0}; second, *n_i_* was taken as the grade classification criterion, and the grade interval was (*n_i_*, *n_i+_*_1_); if the initial comprehensive LES index *L_m_* (*m* = 1, 2, …, 7684) satisfied *n_i_* ≤ *L_m_* ≤ *n_i+_*_1_, then the adjusted composite index *L′_m_* was calculated by a formula (3) and finally, according to *L′_m_*, the natural breakpoint method was used to determine the grading interval.

(3) $${L^{\prime }}_{m}=\left(\frac{L_{m}-n_{i}}{n_{i+1}-n_{i}}\right)\times 0.1$$

### 3.2. Land Use Pattern Simulation

Cellular automata (CA) is a spatial dynamic model with discrete time, space, and state, which has the ability to simulate the spatiotemporal evolution process of complex systems [36,37]. The model consists of four parts—cell, state, neighborhood range, and transfer rule—and the future state of each cell is determined by the current state of the cell, the state of the neighboring cells, and the transfer rule [38]. The Markov model is a method used to predict the probability of a future occurrence of a variable based on its current state, with its distinctive feature being that the future state of things depends only on the current state. This model is widely used in land use/cover change (LUCC) research [39].

The Markov model focuses on the prediction of quantity and cannot show the spatial dynamics of land use change, while the CA model can spatially show the spatial and temporal evolution of complex systems. The CA–Markov model combines the respective advantages of the CA model and the Markov model, solving the key problem of simultaneous simulation of land use temporal dynamics and spatial patterns, and thus can better simulate the future spatial and temporal patterns of land use [40]. The model equation is as follows:

(4) $$s_{ij}\left(t+1\right)=f\left[s_{ij}\left(t\right),h_{ij}\left(t\right),Q\right]$$

where *s_ij_* (*t* + 1) and s*_ij_* (*t*) represent the state of the cell with location (*i*, *j*) at time *t* + 1 and *t*, respectively; *h_ij_* (*t*) represents the state of the neighborhood of the cell at location (*i*, *j*); *Q* represents the suitability atlas; and *f* represents the conversion rule.

The CA–Markov module in IDRISI Selva 17.0 software was used to simulate the future land use pattern of Guangzhou. The main steps included creating a Markov transfer probability matrix, setting the spatial transformation rules, and constructing the suitability atlas and model calibration. The detailed processes are shown below.

#### 3.2.1. Markov Transfer Probability Matrix Creation

The Markov module in IDRISI was used to calculate the land use conversion probabilities for 2000–2010 and 2010–2020 based on the current land use data for 2000, 2010, and 2020, respectively. The conversion probability for 2000–2010 was used to predict the amount of land use in 2020. The conversion probability for 2010–2020 was used to predict the land use pattern for 2030.

#### 3.2.2. Spatial Transformation Rules Settings

Whether a region attaches importance to ecological conservation will have an important impact on the local future land use pattern. In the CA–Markov model, the future land use pattern is mainly determined by the transfer probability matrix and the suitability atlas, with the transfer probability matrix influencing the quantity of each type of land use, and the suitability atlas influencing the change direction of each type of land use. By setting different transformation rules, the number and direction of future land evolution can be changed, and different utilization patterns can be presented.

In our study, two scenarios were set: a natural development (ND) scenario and an ecological protection (EP) scenario. The conversion rules in the different scenarios were as follows: the natural development scenario does not set the important ecological zone restriction; the ecological protection scenario sets the ecological core zone as a restriction factor and prohibits the expansion of construction land into the ecological core zone; and LESI is set as the constraint factor to realize the simulation of future land use pattern under ecological constraints by reconstructing the suitability atlas.

#### 3.2.3. Construction of Suitability Atlas

Multi-criteria evaluation (MCE) is commonly used for the generation of suitability atlases, and the suitability evaluation criteria include restriction factors and constraint factors. For the k-type land, the restriction factors were the k-type land, permanent basic farmland, water areas, and urban land in the base period, and the ecological core zone in 2020 was added to the construction land restriction factors in the ecological protection scenario. The constraint factors included natural constraint factors and socio-economic constraint factors, and the suitability images of each land use were collected by overlaying the restriction factors and constraint factors in IDRISI using the weighted linear combination method (WLC).

##### Constraint Factors Selection and Weight Determination

Binary logistic regression (BLR) is a nonlinear statistical method for the regression analysis of binary dependent variables [41]. In our study, the land use type was used as the classification dependent variable, and the dependent variable was assigned a value of 1 when the raster was a certain land use type, and 0 otherwise. Based on the availability of data, eight driving factors were selected, including population (POP), distance to river (DR), distance to rural settlement (DRS), distance to highway (DH), distance to town (DT), slope (SLO), elevation (ELE), and LESI, according to the relevant scholars’ studies [37,38,42], and LESI was only included in the ecological protection simulation scenario. The dependent variables and independent variables were tested for collinearity in SPSS. The results of variance inflation factor (VIF) were 1.203–3.517, and the range of toleration (TOL) was 0.284–0.883. There was no collinearity between each factor, and binary logistic regression could be performed [43]. Based on the Create Random Points tool in ArcGIS, 5% of the number of 0 and 1 rasters assigned to each type of land were randomly selected to generate sample points. The BLR model was used to analyze the driving factors of land use change in 2000–2010 (T1) and 2010–2020 (T2), and the weights of constraint factors were determined (Table 3).

#### 3.2.4. Model Calibration

With the 2010 image as the initial image, the Markov transfer matrix for 2000–2010 and the suitability atlas were input, the number of CA cycles was set to 10, and the CA filter was set to 5 × 5 to simulate the land use pattern in 2020. The Kappa coefficient was used to test the consistency between the simulated and actual land use patterns, which was calculated as follows:

(5) $$Kappa=\frac{p_{0}-p_{e}}{1-p_{e}}$$

where *p*_0_ represents the actual consistent rate, and *p_e_* represents the desired consistent rate. Comparing the simulation results with the actual land use map in 2020, the overall accuracy of the predicted six land types is 88.35%, the Kappa coefficient is 0.817, and the Kappa coefficient is greater than 0.75, indicating that the simulation of the future land use pattern of Guangzhou by applying the CA–Markov model has high accuracy and a good simulation effect, and it is feasible to realize the simulation of the future land use scenario based on the model.

## 4. Results

### 4.1. LES Pattern

**Figure 4:**
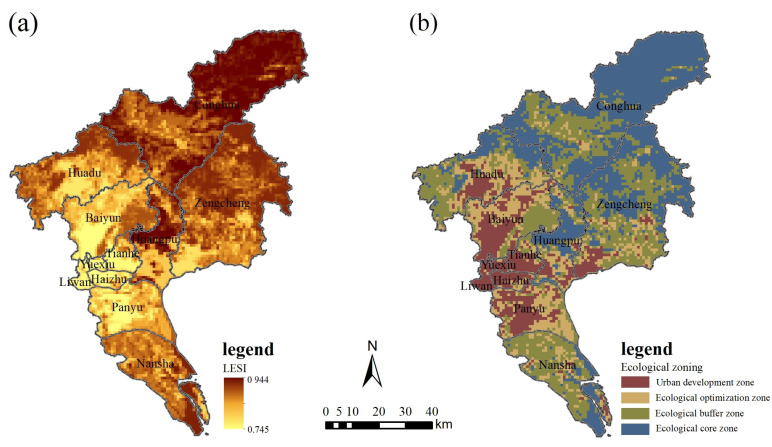
Land ecological security pattern in Guangzhou: (**a**) LESI; (**b**) ecological zoning.

Combined with the improved classification method, the ecological security pattern in Guangzhou was divided into four categories: ecological core zone (ECZ, LESI > 0.915), ecological buffer zone (EBZ, 0.889 < LESI ≤ 0.915), ecological optimization zone (EOZ, 0.855 < LESI ≤ 0.889), and urban development zone (UDZ, 0.745 ≤ LESI ≤ 0.855). The range of LESI in Guangzhou is 0.745–0.944, with an average index of 0.892, indicating that the land security of Guangzhou was at a relatively safe level in 2020 and still needs continuous improvement. The average indices of ECZ, EBZ, EOZ, and UDZ are 0.922, 0.899, 0.881, and 0.845, respectively, and the level of land ecological security in different subregions is highly variable. Structurally, the ecological core zone is 2718.99 km^2^, accounting for 37.53% of the total area, and is mainly composed of woodland, farmland, and water areas. These regions are important ecological barriers in Guangzhou, with less development and good ecological conditions. The urban development zone is 1041.51 km^2^, accounting for 14.37% of the total area, mainly consisting of construction land, and the land ecological security is greatly threatened. The ecological optimization zone area is 1228.88 km^2^, accounting for 16.96% of the total area, and is mainly composed of farmland and construction land, with poor ecological security. The ecological buffer zone area is 2256.40 km^2^, accounting for 31.14% of the total area, mainly consisting of farmland and woodland, with relatively high ecological security. Spatially, the land ecological security pattern differentiation of Guangzhou is obvious, showing regional agglomeration and hierarchical features (Figure 4). The ecological core zone is widely concentrated in the northern part of Conghua District, which is mainly composed of mountainous and water areas, with a high forest coverage rate and lower amounts of human interference, and is an important ecological protection area. In addition, there are many contiguous ecological core areas in the north of Zengcheng District, the middle of Huangpu District, and the south of Nansha District. The urban development zone is mainly concentrated in the central and western areas, consisting of the central cities and towns of Guangzhou. It is an important production and living space, is densely populated, and is in urgent need of ecological improvement. The ecological optimization zone is mostly centered around the urban development zone and is scattered. The ecological buffer zone is mostly concentrated in the central part of Conghua District and Nansha District and the south-central part of Zengcheng District. Overall, the ecological security situation around the town and in industrial areas is poor, and the ecological security level of the ecological conservation areas and the agricultural agglomeration areas is high, with strong spatial heterogeneity.

### 4.2. Analysis of Scenario Simulation Results

#### 4.2.1. Analysis of Land Use Structure Change

Table 4 shows the land use changes in the 2020 and 2030 ND and EP scenarios. In 2020, the total area of farmland, woodland, and construction land was 6559.20 km^2^, accounting for 90.52% of the total area, while grassland, water areas, and unused land accounted for only 9.48%. The prediction results show that in the ND scenario, the construction land increases rapidly, with an increase of 454.58 km^2^, from 21.28% in 2020 to 27.55%. The grassland, water area, and unused land increase by 26.62, 16.05, and 0.17 km^2^, respectively. The farmland and woodland decrease by 270.26 and 227.16 km^2^, respectively. In the EP scenario, the farmland and unused land decrease by 259.75 km^2^ and 0.80 km^2^, respectively. The increase area of woodland is the largest, at 109.88 km^2^, followed by the construction land, which increases by 85.48 km^2^, and the grassland and water area increase less with 47.42 and 17.77 km^2^, respectively. Compared with the ND scenario, the EP scenario shows the largest increase in woodland with 337.04 km^2^, followed by grassland and farmland with an increase of 20.80 and 10.51 km^2^, respectively, while the water area is more stable, increasing by only 1.72 km^2^. The area of construction land decreases by 369.10 km^2^, and the growth rate becomes significantly slower. In the ecological protection scenario, we reduced the occupation of farmland, woodland, grassland, and water area by limiting the expansion of construction land into the ecological core zone, resulting in the main ecological land being effectively protected and the level of regional ecological security significantly improving.

#### 4.2.2. Analysis of Spatial Changes in Land Use

**Figure 5:**
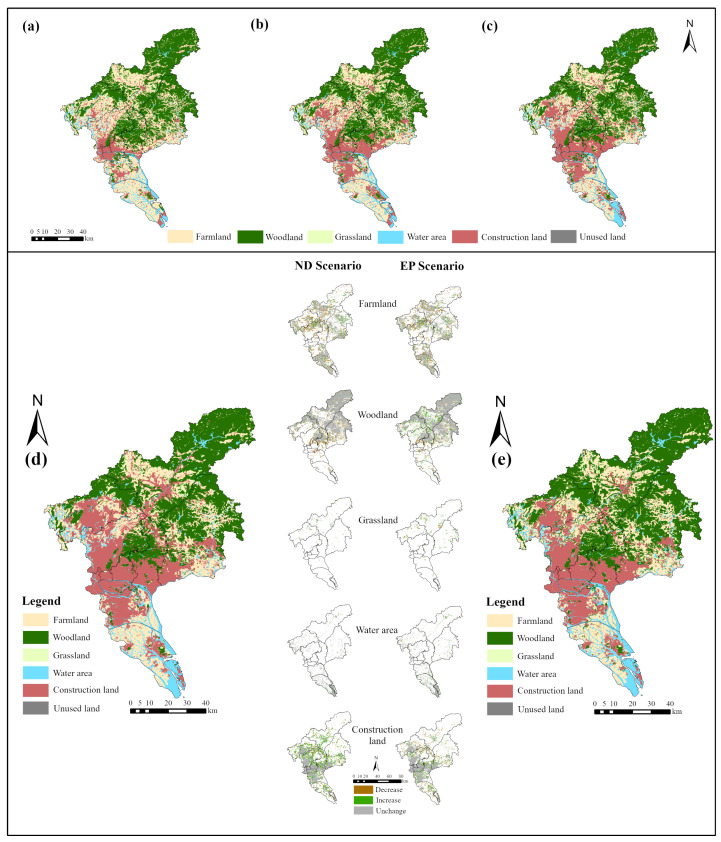
Land use pattern at different time points: (**a**) 2000; (**b**) 2010; (**c**) 2020; (**d**) 2030-ND scenario; (**e**) 2030-EP scenario.

Figure 5 shows the land use pattern for 2030 under the ND and EP scenarios. Spatially, the construction land in both scenarios is mainly distributed in the central region of Guangzhou, and both show outward expansion around the town. The woodland is concentrated in high altitude areas such as Conghua District and the northern part of Zengcheng District, and the reduction area is mainly concentrated in Panyu District, the western part of Conghua District, and the edge of towns in Zengcheng District. The farmland is concentrated in Nansha District, the northern part of Baiyun District, the western part of Conghua District, and the south-central part of Zengcheng District. The grassland distribution is more fragmented, mainly concentrated in the northern part of the study area. The water area is mostly concentrated in the southern part of Nansha District and is scattered elsewhere.

The spatial change in the five main land use types from 2020 to 2030 is shown in Figure 5. In the ND scenario, the expansion of construction land areas is mostly concentrated around the cities and towns, mainly distributed in Huadu District, Baiyun District, Huangpu District, and Zengcheng District, with rapid economic growth. Compared with the ND scenario, in the EP scenario, the expansion of construction land is restricted, and the expansion areas are mainly concentrated around the central urban areas, with less increase in the remaining areas. The two scenarios show different spatial trends in woodland. In the ND scenario, woodland decreases more than increases, and the increase areas are scattered, while in the EP scenario, woodland increases significantly due to the conversion conditions and is mainly concentrated in the central part of Huadu District, Baiyun District, and areas with a high ecological security level, such as Huadu District. In the EP scenario, farmland is occupied at a slower rate in Huangpu District and Huadu District, and the rest of the areas have less variation. The grassland areas show more increase, and the increase areas are mainly concentrated in the eastern part of Zengcheng District and the northern part of Conghua District.

### 4.3. Land Use Pattern Incorporating LES

**Figure 6:**
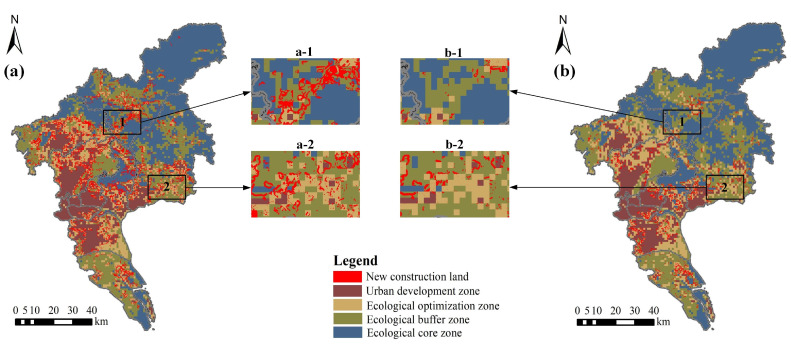
Distribution map of new construction land in different ecological zones: (**a**) ND scenario; (**b**) EP scenario.

The areas of different types of land within different ecological zones in the two scenarios are shown in Table 5. Compared with the ND scenario, the woodland, grassland, and water areas within the ecological core zone increase by 98.33, 16.45, and 16.72 km^2^, respectively, in the EP scenario, while the farmland, woodland, and grassland areas within the ecological buffer zone increase by 30.51, 94.52, and 14.91 km^2^, respectively, and the woodland areas within the ecological optimization zone increase by 146.71 km^2^. To further analyze the expansion of construction land, the new construction land within different ecological zones was further extracted (Figure 6). It was found that the new construction land is mainly distributed in the ecological buffer zone as well as in the ecological optimization zone in the ND scenario; while in the EP scenario, all new construction land is distributed outside the ecological core zone, mainly around the central cities and towns. These results show that the EP scenario has a better effect on limiting the expansion of construction land and maintaining ecological security. They also reveal that the land use pattern simulated based on the EP scenario is more conducive to maintaining regional ecological security than the land use pattern simulated by the ND scenario in the context of ecological civilization construction in China.

## 5. Discussion

### 5.1. The Simulation Model Integrating LES

The CA–Markov model is an effective method that can be used to achieve future land use prediction and can be combined with a variety of models, such as Random Forest [44], DEMATEL-ANP [45], and Logistic Regression [23], to realize the simulation of geospatial patterns. In our study, the CA–Markov model integrating BLR and MCE was constructed for the simulation of future land use patterns, and the results show that compared with the ND scenario, the expansion of construction land in the EP scenario is slower; the woodland area, water area, and other ecological land areas increase; and the level of ecological security is significantly improved. The ecological core zone is important for maintaining biodiversity and protecting ecological security. Our study takes the land ecosystem as the core, delineates ecological zoning through land ecological security assessment from a sustainable perspective, and determines the ecological core zone. Compared with the method of determining the ecological core zone based on landscape pattern analysis and ecosystem service value accounting [11,46], ecological zoning based on land ecological security assessment takes into account the influence of human activities and the natural environment, as well as considering socio-economic factors, avoiding the disadvantages of determining zoning based on a single natural type of factor, and thus ignoring the influence of economic and social development on future land use trends. In terms of setting ecological protection scenarios, our study treats core ecological zones as restricted conversion areas and prohibits the conversion of construction land, similar to related studies [47]. However, instead of simply setting spatially restricted areas, our study takes into account the suitability of various land types in different ecological zones, sets the LESI as the constraint factor, and realizes the simulation of land use patterns in the EP scenario by reconstructing the suitability atlas. In terms of the selection of the driving factors of land use change, our study determined the significant driving factors and weights for different periods based on binary logistic regression (BLR), which is more objective and applicable compared with other studies that selected driving factors qualitatively and determined weights subjectively based on an analytic hierarchy process (AHP) [48].

### 5.2. LES Evaluation

Our study comprehensively evaluated the land ecological security situation in Guangzhou and divided the ecological security zones. The results showed that the spatial differentiation of the land ecological security pattern was obvious, showing regional agglomeration and stratification characteristics, consistent with the conclusions reached by related studies [49,50]. We took ecological resource endowment as the starting point, integrated the interrelationship between human activities and the natural environment, and established a comprehensive index system based on resource and environmental indicators and socio-economic indicators through the PSR model, avoiding the influence of a single type of indicator on the evaluation results [33,51]. Although current studies mostly use administrative districts as evaluation units with coarse scales [52], we used a kilometer grid as the evaluation unit to further reveal the spatially heterogeneous characteristics of the land ecological security of the study area, facilitating the spatial refinement of the evaluation results. We used the catastrophe progress method to evaluate the land ecological security condition, overcoming the reliance on weights such as the comprehensive index method [34], considering the importance of ranking the indicators, reducing the influence of weight assignment on the evaluation results, and improving the accuracy and objectivity of the evaluation results. Considering that the results calculated by the catastrophe progression method are generally high and concentrated, we adopted an improved method to adjust the LESI index and combined it with the natural breakpoint method to determine the classification threshold, which is different from the method that converts the conventional means of equal division into the membership value to determine the classification threshold [28,53]. This method can highlight the difference between the results, which is conducive to the delineation of ecological zoning.

### 5.3. Implications for Future Land Use and Management

The land use simulation research framework integrated with land ecological security can provide a scientific reference for identifying the ecological security pattern, determining the key ecological regions, formulating land use and protection policies, and improving territorial spatial planning. Based on the current ecological security pattern and the future land use simulation, future land use optimization strategies are proposed. First, Guangzhou has a strong spatial heterogeneity in ecological security levels and can be divided into three types: the northern ecological conservation zone, the central urban built-up area, and the southern ecological regulation zone, based on the spatial relationship and the future land use trends. Considering the significant differences in regional ecological security level, we recommend that a zoning strategy should be implemented. For example, the northern ecological conservation zone should be focused on protection, with ecological restoration and treatment strengthened and human activities controlled; the central urban built-up zone should be treated more vigorously, with the scope of the zone reasonably controlled; and the ecological balance of the southern ecological regulation zone should be maintained, with ecological agriculture actively developed and corresponding policies formulated to limit the excessive expansion of construction land. Second, we predict that the construction land will increase in both scenarios. In the ND scenario, construction land expands more in areas with faster economic growth, while in the EP scenario, construction land is significantly limited in the northern and central parts of the study area due to restrictions on its expansion, with new construction land being mainly concentrated around the central cities and towns. Both scenarios show a spatial trend of the outward expansion of construction land from the central cities and towns, which is also consistent with the current spatial expansion trajectory of most economically developed cities in China [54]. In practice, since the government has planned and controlled the new construction land, and the central government has increasingly emphasized the balance between ecological protection and economic development, the land use pattern in the ecological protection scenario is more conducive to maximizing the overall efficiency of land use. Compared with occupying ecological land in order to realize the expansion of construction land, the process of implementing the renewal of existing construction land and promoting its intensive use is more in line with the current and future urban development trends [55,56]. In future development, it is recommended that the urban development boundary be strictly controlled, the existing construction resources be fully utilized, construction potential be explored, and the occupation of ecological land causing ecological imbalance be avoided.

### 5.4. Limitations and Outlook

Although our study has made contributions to research in related fields, there are still some limitations. The first limitation is that the index system for evaluating land ecological security is not comprehensive enough. Land ecological security is defined as the state in which land ecosystems can maintain their structure and function without, or with few, threats and can also meet the needs of humans for survival and development [57]. This is not only related to natural and socio-economic factors, but planning, policies, and people’s environmental awareness are also factors that affect land ecological security; however, due to the limitation of data acquisition and quantification, such factors were not included in our study. Planning and policies have externalities, which are not suitable for microscopic land ecological security evaluation, and the factors of intention and behavior can be obtained through investigation [58]. The second limitation is the limited scenario setting. Our study focuses on the simulation of land use patterns in the context of an ecological civilization, so only two scenarios, natural development and ecological protection, were set. However, other scenarios, such as over expansion [59] and farmland protection [60], were often set. In the future, land use should be integrated with regional development goals to find a balance between ecological protection, food security, and economic development, so as to achieve maximum benefits. The third limitation is that the driving factors of land use change are not extensive enough. In our study, more natural factors were selected. In fact, land use change is the result of the interaction of various factors. However, due to the influence of data scale, less consideration was given to socio-economic factors. Although some socio-economic data, such as population and GDP, can be rasterized and used in land use simulation [27,42], there are few types of such data that can be spatialized, and the accuracy needs to be improved. Generally, socio-economic data are often obtained through surveys or local governments, and their applicability is closely related to the research units. If socio-economic data at a smaller scale can be obtained, they can better support the determination of driving factors for land use change. Exploring how to realize the integration of socio-economic and grid data at different scales, so as to determine more comprehensive and systematic driving factors, is also an important research direction for land use simulation in the future.

## 6. Conclusions

The urbanization process, which is dominated by the expansion of land for construction in China, has greatly contributed to social and economic development, but it has also caused irreversible damage, and if this process continues, it will seriously affect the sustainable development of the land ecosystem. Therefore, it is of great significance to explore the future land use pattern under the constraints of ecological security to guide practical activities. Our research takes the sustainable development of the land ecosystem as the core, evaluates the level of land ecological security based on the PSR model and the CPM, determines the ecological security zoning, and integrates the ecological security evaluation results into the CA–Markov model incorporated with MCE and BLR to construct the simulation framework of future land use. Taking Guangzhou as an example, we explore the future land use pattern under the two scenarios of natural development (ND) and ecological protection (EP). The simulation results show that the simulation accuracy of the framework is high, and it can realize the simulation of future land use patterns. Under the EP scenario, the increase of construction land slows down, important ecological regions are protected, and the land use pattern is more reasonable. At present, there is a contradiction between economic development and ecological protection in many cities and regions. The key to solving such problems is to reasonably determine the ecological zoning and prevent it in advance. The future land use simulation framework of land ecological security can determine the important ecological areas. On the basis of emphasizing ecological priority and green development, it is of great significance to optimize the land use pattern and reduce the occupation of ecological space by construction. Although our study takes Guangzhou as the study area, the proposed future land simulation framework can also provide a reference for managers of other cities and regions, especially in economically developed regions, to formulate future land use planning and rationally allocate land resources.

In this study, we focus on exploring future land use patterns under ecological security constraints. Although it can provide guidance for the future development of the region, there are shortcomings, such as the limited scenario setting and data acquisition and a non-comprehensive selection of land ecological security evaluation indicators and land use change driving factors. Therefore, future research should explore the integration of socio-economic data with geospatial data to identify a more comprehensive index system. On the other hand, more comprehensive scenarios should be considered in order to improve the prediction ability of this modelling framework and to assist managers and planners in formulating more comprehensive regulations and policies.

## Figures and Tables

**Table 1:** Catastrophe models and normalization formulas.

Type	Number of Control Variables	Potential Function	Bifurcation Set Equation	Normalization Formula
Fold model	1	$D\left(x\right)=x^{3}+ax$	$a=-3x^{2}$	$x_{a}=\sqrt{a}$
Cusp model	2	$D\left(x\right)=x^{4}+ax^{2}+bx$	$a=-6x^{2}$ $,b=8x^{3}$	$x_{a}=\sqrt{a}$ $,x_{b}=\sqrt[{3}]{b}$
Swallowtail model	3	$D\left(x\right)=x^{5}+ax^{3}+bx^{2}+cx$	$a=-6x^{2}$ $,b=8x^{3},$ $c=3x^{4}$	$x_{a}=\sqrt{a}$ $,x_{b}=\sqrt[{3}]{b}$ $,x_{c}=\sqrt[{4}]{c}$
Butterfly model	4	$D\left(x\right)=x^{6}+ax^{4}+bx^{3}+cx^{2}+dx$	$a=-10x^{2}$ $,b=20x^{3}$ $,c=-15x^{4}$ $,d=4x^{5}$	$x_{a}=\sqrt{a}$ $,x_{b}=\sqrt[{3}]{b}$ $,x_{c}=\sqrt[{4}]{c}$ $,x_{d}=\sqrt[{5}]{d}$

**Table 2:** Index system of LES evaluation.

Target Layer	Criteria Layer	Factor Layer	Index Layer	Unit	Weight	Attribute
LES (A)	Pressure (B1)	Environmental pollution (C1)	Per farmland chemical fertilizer (D1)	kg·ha^−1^	0.005	Negative
			Per farmland pesticide (D2)	kg·ha^−1^	0.012	Negative
		Population growth (C2)	Population density (D3)	P·km^−2^	0.034	Negative
			Population growth rate (D4)	‰	0.029	Negative
		Urban expansion (C3)	Proportion of construction (D5)	%	0.074	Negative
			Urbanization rate (D6)	%	0.021	Negative
	State (B2)	Environmental quality (C4)	Proportion of woodland (D7)	%	0.082	Positive
			Proportion of water area (D8)	%	0.138	Positive
		Economic condition (C5)	Economic density (D9)	yuan·km^−2^	0.091	Negative
			Per capita GDP (D10)	yuan·P^−1^	0.041	Negative
		Level of resource reserves (C6)	Per capita farmland (D11)	ha·P^−1^	0.045	Positive
			Per capita public green space (D12)	ha·P^−1^	0.024	Positive
			Per capita land reserve resources (D13)	ha·P^−1^	0.074	Positive
	Response (B3)	Pollution treatment (C7)	Attainment rate of the industrial wasted water discharge (D14)	%	0.015	Positive
			Disposal rate of household garbage (D15)	%	0.009	Positive
		Economic input level (C8)	Proportion of tertiary industry value in GDP (D16)	%	0.072	Negative
			Proportion of environment protection investment in GDP (D17)	%	0.085	Positive
		Engineering governance (C9)	Newly added soil erosion control area (D18)	ha	0.043	Positive
			Afforestation renewal area (D19)	ha	0.106	Positive

**Table 3:** Constraint factors and weights.

Land Use Type	Period	Constraint Factors							
		POP	DR	DRS	DH	DT	SLO	ELE	LESI
Farmland	T1	0.231 (−)	0.206 (−)	0.141 (−)	0.107 (+)	0.188 (+)	0.079 (−)	0.048 (−)	—
	T2-ND	0.234 (−)	0.150 (−)	0.140 (−)	0.114 (+)	0.181 (+)	0.093 (−)	0.088 (−)	—
	T2-EP	0.210 (−)	0.134 (−)	0.125 (−)	0.102 (+)	0.162 (+)	0.083 (−)	0.078 (−)	0.106 (−)
Woodland	T1	—	0.114 (+)	0.123 (+)	0.103 (+)	0.117 (+)	0.204 (+)	0.339 (+)	—
	T2-ND	—	0.112 (+)	0.130 (+)	0.111 (+)	0.128 (+)	0.226 (+)	0.293 (+)	—
	T2-EP	—	0.101 (+)	0.117 (+)	0.101 (+)	0.115 (+)	0.204 (+)	0.265 (+)	0.097 (+)
Grassland	T1	0.167 (−)	0.163 (+)	0.158 (+)	0.146 (+)	0.170 (+)	0.196 (+)	—	—
	T2-ND	0.192 (−)	0.184 (+)	0.148 (+)	—	0.161 (+)	0.169 (+)	0.146 (+)	—
	T2-EP	0.171 (−)	0.164 (+)	0.131 (+)	—	0.142 (+)	0.149 (+)	0.129 (+)	0.114 (+)
Water area	T1	0.144 (−)	0.100 (−)	0.184 (+)	0.159 (+)	0.183 (+)	0.127 (−)	0.103 (−)	—
	T2-ND	—	0.117 (−)	0.224 (+)	0.186 (+)	0.222 (+)	0.142 (−)	0.109 (−)	—
	T2-EP	—	0.099 (−)	0.190 (+)	0.159 (+)	0.148 (+)	0.122 (−)	0.093 (−)	0.189 (+)
Construction land	T1	0.153 (+)	0.220 (+)	0.097 (−)	0.149 (−)	0.093 (−)	0.170 (−)	0.118 (−)	—
	T2-ND	0.213 (+)	0.245 (+)	0.126 (−)	0.169 (−)	0.116 (−)	—	0.131 (−)	—
	T2-EP	0.165 (+)	0.115 (+)	0.106 (−)	0.142 (−)	0.101 (−)	0.153 (−)	0.123 (−)	0.095 (−)
Unused land	T1	0.303 (−)	0.108 (−)	0.123 (+)	0.125 (+)	0.146 (+)	—	0.195 (+)	—
	T2-ND	0.282 (−)	0.138 (−)	0.140 (+)	0.133 (+)	0.142 (+)	—	0.165 (+)	—
	T2-EP	0.253 (−)	0.124 (−)	0.126 (+)	0.120 (+)	0.127 (+)	—	0.149 (+)	0.101 (+)

Note: “—” is non-significantly correlated or not considered and is not included in the constraint factor; “(−)” indicates negative correlation; “(+)” indicates positive correlation.

**Table 4:** Land use change in the ND and EP scenarios.

Land Use Type	Area/km^2^			Difference/km^2^		Percentage/%	
	2020	2030-ND	2030-EP	2030-ND	2030-EP	2030-ND	2030-EP
Farmland	1998.29	1728.03	1738.54	−270.26	−259.75	−13.52	−13.00
Woodland	3019.18	2792.02	3129.06	−227.16	109.88	−7.52	3.64
Grassland	103.79	130.41	151.21	26.62	47.42	25.65	45.69
Water area	580.71	596.76	598.48	16.05	17.77	2.76	3.06
Construction land	1541.73	1996.31	1627.21	454.58	85.48	29.49	5.54
Unused land	2.08	2.25	1.28	0.17	−0.80	8.17	−38.46

**Table 5:** Land use area and proportion in ecological zones.

Land Use Types	Scenarios	Categories	Ecological Zones				Total
			UDZ	EOZ	EBZ	ECZ	
Farmland	ND	Area/km^2^	50.29	476.94	1026.80	174.00	1728.03
		Proportion/%	2.91%	27.60%	59.42%	10.07%	100.00%
	EP	Area/km^2^	42.26	511.03	1057.31	127.94	1738.54
		Proportion/%	2.43%	29.39%	60.82%	7.36%	100%
Woodland	ND	Area/km^2^	4.73	66.84	516.94	2203.51	2792.02
		Proportion/%	0.17%	2.39%	18.51%	78.92%	100%
	EP	Area/km^2^	2.21	213.55	611.46	2301.84	3129.06
		Proportion/%	0.07%	6.82%	19.54%	73.56%	100%
Grassland	ND	Area/km^2^	2.68	27.87	61.07	38.79	130.41
		Proportion/%	2.06%	21.37%	46.83%	29.74%	100%
	EP	Area/km^2^	2.35	17.64	75.98	55.24	151.21
		Proportion/%	1.55%	11.67%	50.25%	36.53%	100%
Water area	ND	Area/km^2^	31.44	107.44	264.66	193.22	596.76
		Proportion/%	5.27%	18.00%	44.35%	32.38%	100%
	EP	Area/km^2^	32.34	115.68	240.52	209.94	598.48
		Proportion/%	5.40%	19.33%	40.19%	35.08%	100%
Construction land	ND	Area/km^2^	952.37	549.60	384.95	109.39	1996.31
		Proportion/%	47.71%	27.53%	19.28%	5.48%	100%
	EP	Area/km^2^	962.35	370.79	270.17	23.90	1627.21
		Proportion/%	59.14%	22.79%	16.60%	1.47%	100%
Unused land	ND	Area/km^2^	0.00	0.19	1.98	0.08	2.25
		Proportion/%	0.00%	8.44%	88.00%	3.56%	100%
	EP	Area/km^2^	0.00	0.19	0.96	0.13	1.28
		Proportion/%	0.00%	14.84%	75.00%	10.16%	100%

## Data Availability

The data and models generated or used during the study appear in the submitted article.
